# Cost-utility of routine cataract surgery

**DOI:** 10.1186/1477-7525-4-74

**Published:** 2006-09-29

**Authors:** Pirjo Räsänen, Kari Krootila, Harri Sintonen, Tiina Leivo, Anna-Maija Koivisto, Olli-Pekka Ryynänen, Marja Blom, Risto P Roine

**Affiliations:** 1 Helsinki and Uusimaa Hospital Group, Group Administration, P.O.Box 440, 00029 HUS, Helsinki, Finland; 2 Helsinki University Eye Hospital, P.O.Box 220, 00029 HUS, Helsinki, Finland; 3 University of Helsinki, Department of Public Health and Finnish Office for Health Technology Assessment, Helsinki, Finland P.O.Box 41, 00014 Yliopisto, Helsinki, Finland; 4 University of Tampere, School of Public Health, 33014 Yliopisto, Tampere, Finland; 5 University of Kuopio, Department of Health Policy and Management, P.O.Box 1627, 70211 Kuopio, Finland; 6 Academy of Finland, c/o Stakes and Jorvi Hospital, Espoo, Finland, P.O.Box 220, 00531 Helsinki, Finland

## Abstract

**Background:**

If decisions on health care spending are to be as rational and objective as possible, knowledge on cost-effectiveness of routine care is essential. Our aim, therefore, was to evaluate the cost-utility of routine cataract surgery in a real-world setting.

**Methods:**

Prospective assessment of health-related quality of life (HRQoL) of patients undergoing cataract surgery. 219 patients (mean (SD) age 71 (11) years) entering cataract surgery (in 87 only first eye operated, in 73 both eyes operated, in 59 first eye had been operated earlier) filled in the 15D HRQoL questionnaire before and six months after operation. Direct hospital costs were obtained from a clinical patient administration database and cost-utility analysis performed from the perspective of the secondary care provider extrapolating benefits of surgery to the remaining statistical life-expectancy of the patients.

**Results:**

Mean (SD) utility score (on a 0–1 scale) increased statistically insignificantly from 0.82 (0.13) to 0.83 (0.14). Of the 15 dimensions of the HRQoL instrument, only seeing improved significantly after operation. Mean utility score improved statistically significantly only in patients reporting significant or major preoperative seeing problems. Of the subgroups, only those whose both eyes were operated during follow-up showed a statistically significant (p < 0.001) improvement. Cost per quality-adjusted life year (QALY) gained was €5128 for patients whose both eyes were operated and €8212 for patients with only one eye operated during the 6-month follow-up. In patients whose first eye had been operated earlier mean HRQoL deteriorated after surgery precluding the establishment of the cost per QALY.

**Conclusion:**

Mean utility gain after routine cataract surgery in a real-world setting was relatively small and confined mostly to patients whose both eyes were operated. The cost of cataract surgery per quality-adjusted life year gained was much higher than previously reported and associated with considerable uncertainty.

## Background

The society invests in health care without definite knowledge about the health gains produced as systematic assessment of various interventions is usually lacking. This holds especially true for patient values, i.e. the subjective benefits that patients perceive from treatments.

Cataract surgery is a routine intervention, the demand for which is expected to strongly increase as the population is ageing. However, even the present need for cataract surgery is uncertain as suggested by the great variation in cataract operation rates both between countries and, in some countries, within the country [1]. Knowledge on the cost-effectiveness of cataract surgery is thus essential if decisions on health care spending are to be as rational and objective as possible.

In the field of ophthalmology, effectiveness has mostly been measured using disease-specific instruments [2-8]. They have usually demonstrated that cataract operations are effective. The disease-specific instruments, however, do not allow the comparison of cost-effectiveness of different interventions in different medical specialities [9]. This can only be achieved by using generic (non disease-specific) health-related quality of life (HRQoL) instruments that produce a single index number (utility).

Using generic instruments cataract operation has in some studies been associated with an improvement in HRQoL [9-11]. Furthermore, visual acuity and visual disability have been reported to correlate significantly with utilities and it has been suggested that data on visual acuity and disability in large registries could be used to estimate the costs per quality-adjusted life years (QALY) gained by cataract surgery [9].

Several other studies employing widely used generic HRQoL instruments and large patient samples, however, have been unable to detect a significant improvement in perceived utility after cataract operation [2,12,13] and one study even reported that by one year after cataract surgery the scores of all SF-36 dimensions, except for role disability due to mental health problems, were worse than before surgery [14].

Thus the effectiveness and cost-effectiveness in terms of utility of cataract surgery, especially under routine circumstances in a real-world setting, and when compared to other healthcare interventions, still remain undetermined. The aim of our study was to evaluate the cost-utility of cataract surgery, compared to a hypothetical situation of no treatment by studying unselected patients referred by practicing ophthalmologists for a routine cataract operation to a large university clinic because of objective signs of poor visual acuity due to cataract. As we were particularly interested in the effectiveness of the routine practice of providing cataract surgery under everyday conditions, i.e., the standard custom of treating patients in the hospital at the time of the study, selection for surgery was not based on any concise predefined criteria but on individual ophthalmologists' assessment of the patients' subjective seeing problems and objective signs concerning visual acuity and presence of signs of cataract.

## Methods

In the framework of a large trial exploring the feasibility of routine evaluation of cost-utility of secondary health care provided by a referral hospital offering secondary and tertiary health-care services for a population of approximately 1.4 million, we have collected HRQoL data on more than 10000 patients in 10 different medical specialities before and after interventions [15]. The observed change in HRQoL is linked to routinely collected cost data to determine the utility of various interventions. The ultimate goal of the project is to provide decision makers with relevant information for planning of future secondary health care services.

In the field of ophthalmology, 386 patients scheduled for routine cataract operation in the Helsinki University Eye Hospital between August 2002 and June 2003 were invited to participate and to fill in the 15D HRQoL questionnaire. Of them, 88% agreed and returned the baseline questionnaire. A follow-up questionnaire was mailed to all patients having returned the baseline questionnaire approximately six months after the cataract operation. 282 patients (73% of those originally asked to participate) also returned the follow-up questionnaire and were available for analysis. However, seven cases were removed from further analysis because of incomplete data (more than three missing answers on the 15 dimensions of the HRQoL instrument or missing answer to the seeing dimension), 32 patients because they had filled in the baseline questionnaire after the operation, six patients because they had filled in the follow-up questionnaire less than 60 days after the operation of the second eye, seven patients because the eventual intervention performed was more extensive than a plain cataract operation, and 11 patients because their final principal diagnosis was not cataract. Thus 219 cataract patients were available for final analysis.

### Visual acuity

Visual acuity is the measurement of the ability to discriminate two stimuli separated in space at high contrast relative to the background. Clinically, this is measured by asking the subject to discriminate letters of known visual angle. The visual acuity is represented as the reciprocal of the minimal angle of resolution (the smallest letters resolved) at a given distance and at high contrast [16]. Best corrected visual acuity (BCVA) before the operation was determined in both eyes by the widely used Snellen notation at 6 meters.

### Health-related quality of life

Health-related quality of life (HRQoL) was measured by the 15D. It is a generic, 15-dimensional, standardised, self-administered HRQoL instrument that can be used both as a profile and a single index utility score measure [17]. The 15D questionnaire consists of 15 dimensions: moving, seeing, hearing, breathing, sleeping, eating, speech, eliminating, usual activities, mental function, discomfort and symptoms, depression, distress, vitality and sexual activity. For each dimension, the respondent must choose one of the five levels that best describes his/her state of health at the moment (the best level = 1; the worst level = 5). The valuation system of the 15D is based on an application of the multi-attribute utility theory. A set of utility or preference weights, elicited from the general public through a 3-stage valuation procedure, is used in an additive aggregation formula to generate the utility score, i.e. the 15D score (single index number) over all the dimensions. The maximum score is 1 (no problems on any dimension), and minimum score 0 (equal to being dead). In most of the important properties the 15D compares favourably with other instruments of that kind [18-21].

### Cost-utility

The perspective taken for the analysis was that of the secondary health care provider. Direct health care costs were obtained from the Ecomed^® ^clinical patient administration system (Datawell Ltd., Finland), where all costs of treatment of individual patients in the hospital are routinely stored. The cost data was from years 2002–2003, i.e. from the same period as the effectiveness (15D) data, and covered all relevant specialty-related costs including pre- and postoperative outpatient visits to the eye hospital. However, the costs of the visits to the referring ophthalmologists who were usually also responsible for the post-operative re-examination of the patients and prescription of eyeglasses, was not included in the analysis. Indirect costs, like period of disability, were not included.

The HRQoL gain was assumed to last till the end of the remaining statistical life expectancy of each patient based on Life Tables from 2002 from Statistics Finland [22]. Although this is not strictly true as HRQoL of patients tends to deteriorate over the years, this approach is typically used for the calculation of QALYs gained by medical interventions, and dividing mean costs by the mean number of QALYs gained gives an estimate of cost-utility in the form of cost per QALY. As the gain of cataract surgery is anticipated to last for many years, whereas the costs accrued during the study period, the number of QALYs and consequent cost per QALY figures are also reported using a discount rate of 5%.

### Ethical considerations

All patients received routine treatment and were not, besides being asked to fill in the 15D questionnaire and to give a written informed consent, approached in any other way. The study protocol was approved by the Ethical Committee of the Helsinki and Uusimaa Hospital District. The trial has been registered in the Helsinki and Uusimaa Hospital District Clinical Trials Register [23] with the unique trial number 75370.

### Statistical methods

Data were analysed using SPSS for Windows version 11.0 statistical software (SPSS, Inc., Chicago, IL, USA) and the R environment for statistical computing and graphics [24]. The results are given as mean and standard deviation (SD) or as mean and 95% confidence interval (CI) or as median. For continuous variables, the significance of the differences between the groups was analyzed using one-way analysis of variance followed by post-hoc comparisons with independent samples t-test. The significance of the differences between before and after treatment scores was analyzed with Student's paired t-test for dependent samples. Independent samples t-test was used to compare these scores with age- and gender-standardized general population. The relationship of the dimension seeing and visual acuity was assessed by Spearman correlation. Visual acuity data obtained by the Snellen chart were converted to LOGarithm of Minimal Angle of Resolution (LogMAR) units for statistical analysis using the Visual Acuity Conversion Chart [25]. A p-value < 0.05 was considered statistically significant.

One-way sensitivity analyses were performed by varying the discount rate between 1–5%, using the median values of QALY gain and costs, and using the upper and lower values of the 95% CI for the mean differences in treatment effectiveness (HRQoL change) and costs. To assess the degree of uncertainty 10000 re-samples from the original stochastic cost-utility data set were simulated using a bootstrapping technique. Bootstrap results are presented as cost-effectiveness planes and cost-effectiveness acceptability curves.

## Results

Preoperative and six-month follow-up data were available from 219 patients (mean age 71 (11) years, 65% females). The study population comprised three different subgroups: group A: only one eye was operated (n = 87), group B: both eyes were operated during the follow-up (n = 73), and group C: first eye had been operated earlier, now the second eye was operated (n = 59).

**Figure 1:**
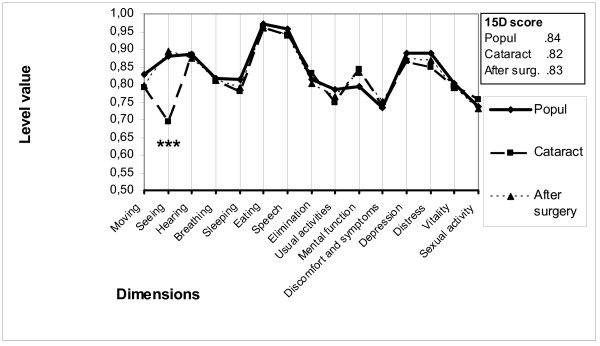
**15D profiles for the study group and age- and sex-matched controls**. Profiles for the operated patients are shown at baseline and six months after cataract surgery. (*** = significant improvement at a p level < 0.001).

Compared to age- and gender-matched general population based on data from a nation-wide survey [26], cataract patients were preoperatively statistically significantly worse off on the dimensions seeing, moving, sleeping, usual activities, depression and distress, but better off on the dimension of mental function. However, the overall utility score did not differ in a statistically significant manner between the general population and the patients (Figure 1).

In the whole sample, the overall utility score showed a statistically insignificant improvement six months after cataract surgery compared to baseline from 0.82 (0.13) to 0.83 (0.14). Of the subgroups, only group B showed a statistically significant improvement in utility from 0.80 (0.13) to 0.83 (0.14), (p < 0.001), whereas HRQoL remained almost constant in group A and deteriorated after cataract surgery in group C (Table 1).

**Table 1:** Health-related quality of life (HRQoL) and cost data in groups A-C

Variable	Group A (n = 87)	Group B (n = 73)	Group C (n = 59)
Mean age, years	69 (12)	70 (12)	75 (10)
Female, %	56	71	71
HRQoL at baseline	0.85 (0.13)	0.80 (0.13)	0.82 (0.11)
HRQoL at 6 months	0.85 (0.14)	0.83 (0.14)	0.81(0.13)
HRQoL difference between baseline and 6 months	0.00 (0.14) p = 0.852	0.03 (0.14) p < 0.001	-0.01 (0.07) p = 0.279
Mean hospital costs at 6 months, €	1318 (184)	2289 (266)	1323 (361)
Mean QALYs gained	0.1605 (0.9421)	0.4464 (1.1966)	-0.0219 (0.7424)
Cost per QALY, €	8212	5128	Can not be established

Values are percentages or means with standard deviations (SD) in parentheses. Group A = patients with only one eye operated; Group B = patients with both eyes operated during follow-up; Group C = patients who had had their first eye operated earlier and now the second eye was operated.

**Figure 2:**
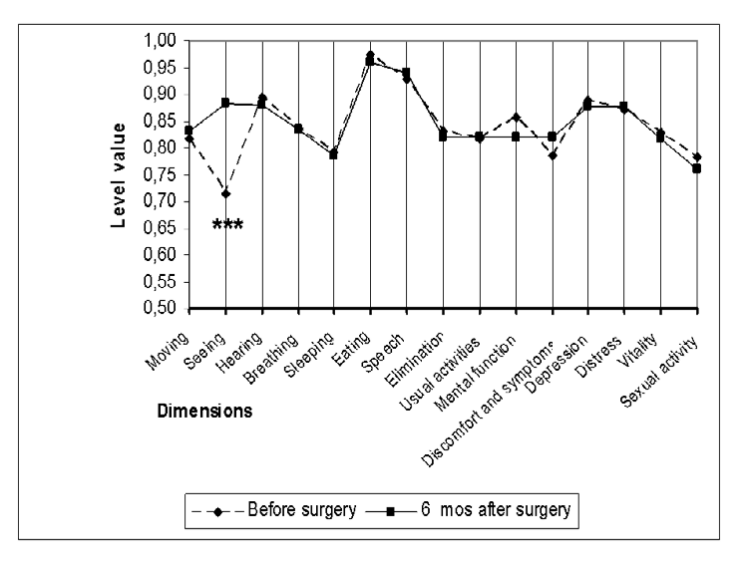
**15D profiles before and six months after cataract surgery in group A**. Group A = patients with only one eye operated. (*** = significant improvement at a p level < 0.001).

**Figure 3:**
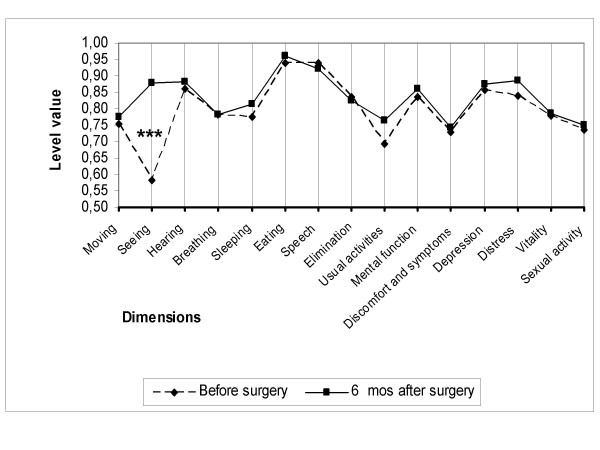
**15D profiles before and six months after cataract surgery in group B**. Group B = patients with both eyes operated during follow-up. (*** = significant improvement at a p level < 0.001).

**Figure 4:**
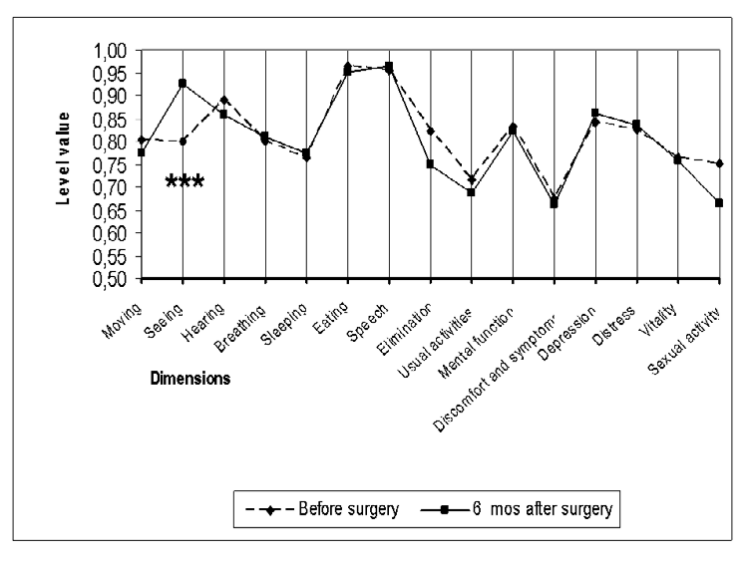
**15D profiles before and six months after cataract surgery in group C**. Group C = patients who had had their first eye operated earlier and now the second eye was operated. (*** = significant improvement at a p level < 0.001).

**Figure 5:**
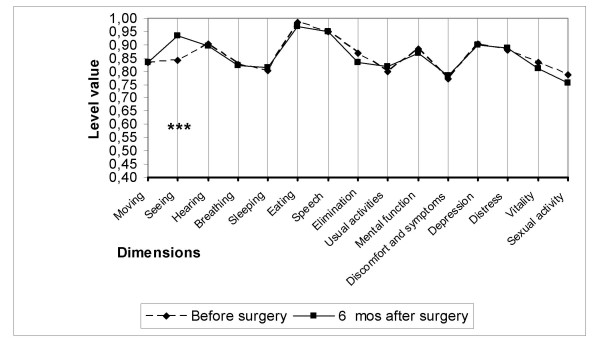
**15D profiles in patients reporting minor preoperative seeing problems**. Patients with minor preoperative seeing problems = levels 1 and 2 of the seeing dimension, (*** = significant improvement at a p level < 0.001).

**Figure 6:**
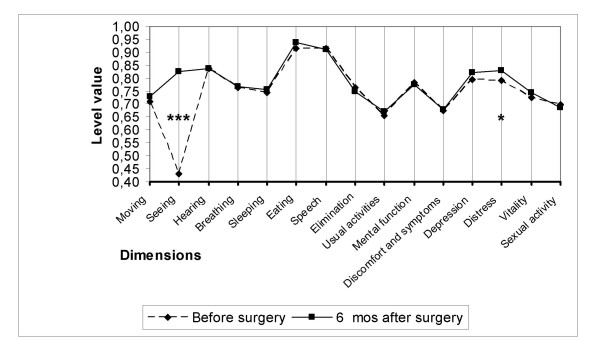
**15D profiles in patients reporting significant preoperative seeing problems**. Patients with significant preoperative seeing problems = levels 3 – 5 of the seeing dimension. (* = significant improvement at a p level < 0.05, *** = significant improvement at a p level < 0.001).

Of the 15 dimensions of the instrument, only the one evaluating seeing improved as a consequence of cataract surgery in all three subgroups (Figures 2, 3, 4). On the five-level seeing dimension of the 15D instrument, 17% of patients reported having no preoperative difficulties in seeing and 47% only minor difficulties (levels 1 and 2 of the seeing dimension). Although those patients experienced a slight improvement in seeing, their mean utility score did not improve as a result of surgery (Figure 5). In patients reporting significant or major preoperative seeing problems (levels 3 to 5 of the seeing dimension), cataract surgery improved seeing (p < 0.001) and distress (p = 0.036), and also had a statistically significant positive effect on the overall utility score which increased from 0.76 (0.14) to 0.78 (0.15)(p = 0.02) (Figure 6).

The mean best corrected visual acuity in the eye to be operated in groups A-C is shown in Table 2 and that of patients with significant preoperative seeing problems compared to those with no or only minor problems in Table 3. The correlation between the best corrected visual acuity (expressed in LogMAR units) in the surgical eye and the subjective level of seeing (the seeing dimension of the HRQoL-instrument) was poor (R = 0.17, p = 0.013). However, the visual acuity of the non-surgical eye correlated fairly well with the seeing dimension of the 15D instrument (R = 0.503, p < 0.001).

**Table 2:** Best-corrected visual acuity in the surgical eye and the non-surgical eye prior to cataract surgery in groups A-C

		Group A (n = 87)	Group B (n = 73)	Group C (n = 59)	Significance
Mean best corrected visual acuity in the surgical eye	Snellen	0.19 (0.14)	0.17 (0.12)	0.24 (0.14)	
	LogMAR	0.98 (0.66)	0.94 (0.49)	0.76 (0.48)	N.S.
Mean best corrected visual acuity in the non-surgical eye	Snellen	0.58 (0.23)	0.28 (0.16)	0.63 (0.25)	
	LogMAR	0.29 (0.22)	0.65 (0.37)	0.25 (0.21)	A vs B p < 0.001 B vs C p < 0.001 A vs C N.S.

Values are means with standard deviations (SD) in parentheses expressed both as Snellen units as well as LOGarithm of Minimal Angle of Resolution (LogMAR) units. Group A = patients with only one eye operated; Group B = patients with both eyes operated during follow-up; Group C = patients who had had their first eye operated earlier and now the second eye was operated.

**Table 3:** Best-corrected visual acuity in the surgical eye and the non-surgical eye prior to surgery in patients reporting minor or significant preoperative seeing problems

		Patients with no or only minor subjective preoperative seeing problems n = 140	Patients with significant subjective preoperative seeing problems n = 79	Significance
Mean best corrected visual acuity in the surgical eye	Snellen	0.21 (0.14)	0.17 (0.12)	
	LogMAR	0.88 (0.59)	0.94 (0.52)	N.S.
Mean best corrected visual acuity in the non-surgical eye	Snellen	0.57 (0.24)	0.35 (0.24)	
	LogMAR	0.30 (0.23)	0.58 (0.39)	p < 0.001

Values are means with standard deviations (SD) in parentheses expressed both as Snellen units as well as LOGarithm of Minimal Angle of Resolution (LogMAR) units. Patients with minor preoperative seeing problems = levels 1 and 2 of the seeing dimension; patients with significant preoperative seeing problems = levels 3 – 5 of the seeing dimension.

Mean (SD) cost of cataract surgery in the whole sample was €1261 (246) per eye operated. In the whole patient sample the cost per QALY gained (assuming that a favourable outcome from cataract surgery lasts till the end of life) was €7947. In subgroup A the cost per QALY was €8212 and in subgroup B €5128, respectively. In subgroup C the cost per QALY gained could not be established as the change in utility was negative.

In one-way sensitivity analysis the cost per QALY was relatively robust against discounting or varying the cost or effectiveness of treatment within the 95% confidence interval observed in the study (Table 4). However, using median values increased the cost per QALY substantially in the group of patients whose first eye had been operated earlier.

**Table 4:** Cost-utility sensitivity analysis

	Patients with only one eye operated n = 87 Group A			Patients with both eyes operated n = 73 Group B			Patients who had had their first eye operated earlier and now the second eye was operated n = 59 Group C		
	Costs	QALY gain	Cost per QALY gained	Costs	QALY gain	Cost per QALY gained	Costs	QALY gain	Cost per QALY gained
Base case analysis using mean values	1318	0.1605	8212	2289	0.4464	5128	1323	-0.0219	Can not be established
Base case analysis using median values	1301	0.0332	39188	2342	0.2989	7835	1195	-0.0234	Can not be established
Sensitivity analysis varying the discount rate for QALYs									
*discount rate 5%*		0.0710	18563		0.2873	7967		-0.0397	Can not be established
*discount rate 3%*		0.0982	13422		0.3377	6778		-0.0375	Can not be established
*discount rate 1%*		0.1390	9482		0.4052	5649		-0.0329	Can not be established
Sensitivity analysis varying treatment effectiveness (QALY gain)									
*upper 95% CI*		0.3613	3648		0.7256	3155		0.1716	7710
*lower 95% CI*		-0.0403	Can not be established		0.1672	13690		-0.2153	Can not be established
Sensitivity analysis varying treatment costs									
*upper 95% CI*	1357		8455	2351		5267	1417		Can not be established
*lower 95% CI*	1279		7969	2227		4989	1229		Can not be established

Base case and sensitivity analyses varying the discount rate between 1–5%, using median values, and using the upper and lower values of the 95% confidence interval (CI) for the mean differences in treatment effectiveness (improvement in health-related quality of life) and costs (QALY = quality-adjusted life year).

**Figure 7:**
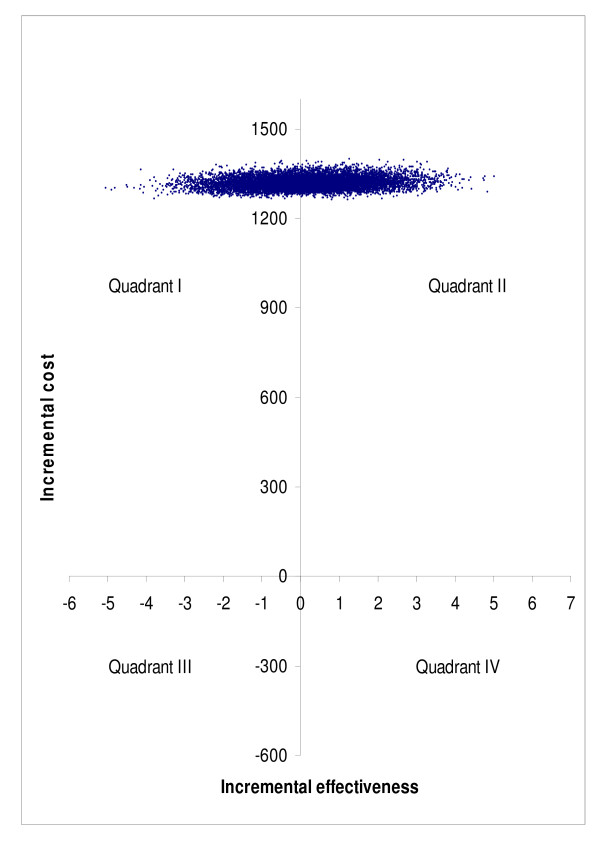
**Representation of cost-effectiveness planes of groups A on basis of bootstrap simulation**. Group A = patients with only one eye operated.

**Figure 8:**
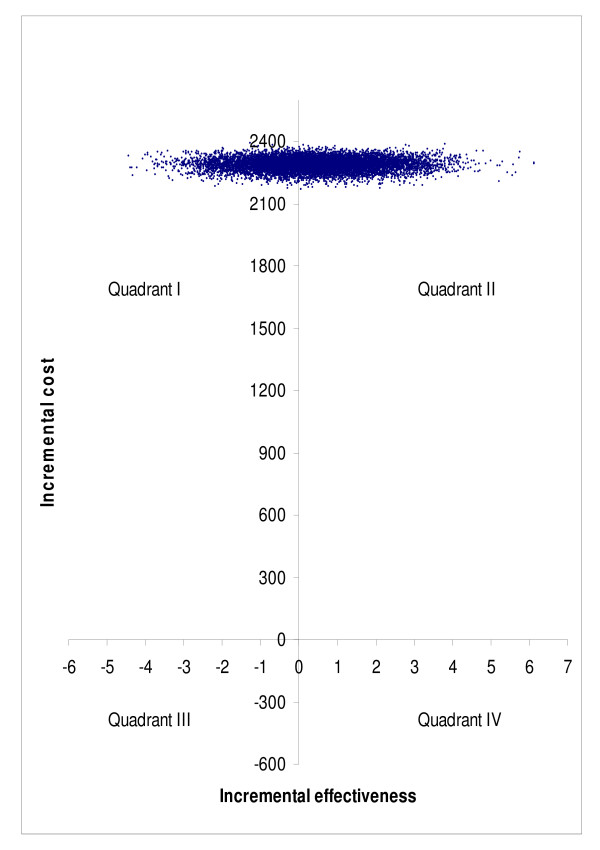
**Representation of cost-effectiveness planes of groups B on basis of bootstrap simulation**. Group B = patients with both eyes operated during follow-up.

**Figure 9:**
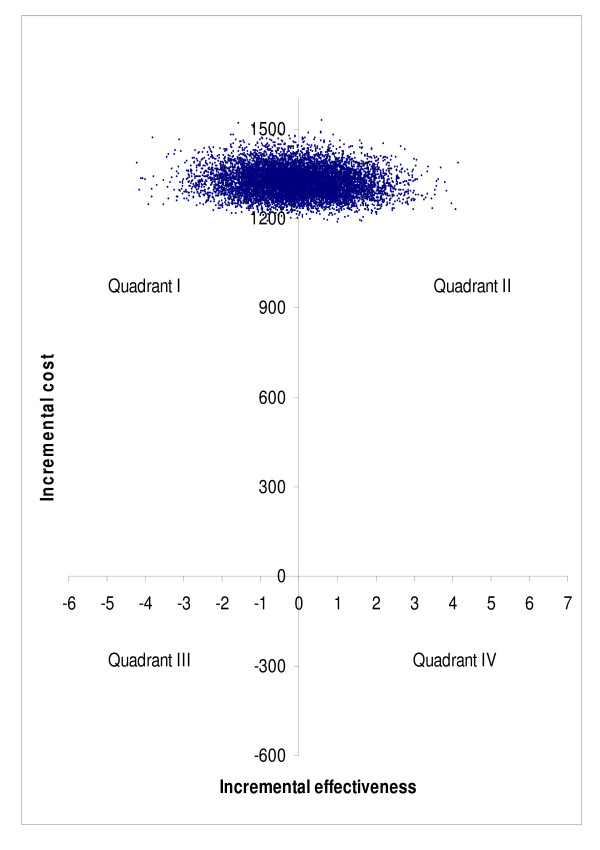
**Representation of cost-effectiveness planes of groups C on basis of bootstrap simulation**. Group C = patients who had had their first eye operated earlier and now the second eye was operated.

**Figure 10:**
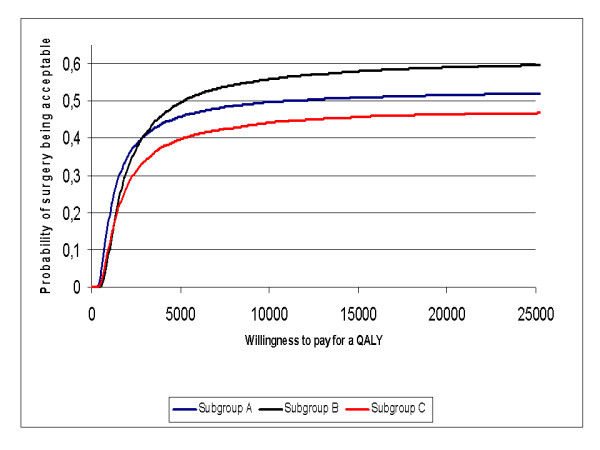
**Cost-effectiveness acceptability curve for groups A-C**. Group A = patients with only one eye operated; Group B = patients with both eyes operated during follow-up; Group C = patients who had had their first eye operated earlier and now the second eye was operated.

Bootstrap simulation suggested that compared to no treatment, surgery was more costly and less effective in 46.4% of simulated cases, and more costly and more effective in 53.6% of simulated cases in subgroup A (quadrant I vs. quadrant II in Figure 7). The corresponding percentages were 37.9% and 62.1% in subgroup B (Figure 8), and 51.1% and 48.9% in subgroup C (Figure 9), respectively. Bootstrap sensitivity analysis also suggested that at a willingness to pay threshold of €20 000 per QALY gained the probability of cataract surgery being acceptable was 51.7% in subgroup A, 59. 0% in subgroup B and 46. 4% in subgroup C (Figure 10).

## Discussion

It has been argued that cataract surgery is one of the most cost-effective procedures in medicine, and that not only the initial cataract surgery is cost-effective, but also restoring binocular vision with second-eye surgery is cost-effective [27]. Previous studies have estimated the cost per QALY gained by first eye cataract surgery to range from $2020 to $4500 [9,28] and that by second eye surgery to be $2727 [27]. Those estimates were, however, based on observed visual acuity data translated into utility values, not actual measurements of HRQoL of individual patients as in our study. Our results, therefore, may better reflect the utility patients gain from a cataract operation. This gain appears to be smaller, at least under everyday settings, than many earlier studies have estimated and, consequently, the cost per QALY gained also significantly higher than previously reported. Besides, bootstrap simulation indicated that the point estimates are associated with a considerable degree of uncertainty.

If we apply to our data the same methodology and assumptions as Kobelt et al. in a cross-sectional study [9] to estimate the utility gain from cataract surgery, the gain in our material is 0.023 and thus almost identical to that estimated by Kobelt et al. (0.028), but more than two-fold compared to that actually observed in this study based on before-after data from routine cataract operations in an everyday setting. Applying the gain calculated in the manner suggested by Kobelt et al. to QALY estimations would bring the cost per QALY gained down to €4958 in our material, i.e. much closer to the estimate of US$5000 by Kobelt et al. (undiscounted). This indicates clearly, how important it is to elicit the utility gain from actual before-after measurements of patients rather than to estimate it indirectly from cross-sectional data.

One explanation for the modest impact of cataract surgery on utility in our sample is the fact that approximately two thirds of the patients reported that they had no or only minor subjective seeing problems prior to the operation. This, despite the fact that best corrected visual acuity in the surgical eye was generally relatively poor. However, in many of the cases the poor visual acuity in the surgical eye was compensated by a reasonable visual acuity in the non-surgical eye. As the post-operative re-examination of the patients and prescription of eyeglasses was in the majority of the cases performed by the referring ophthalmologists, we were unfortunately unable to collect post-operative visual acuity data but have no reason to doubt that it would not have improved in most of the patients.

Another reason for the modest impact of cataract surgery on HRQoL could be the mixed patient sample of a university clinic. About one third of our patients had a secondary ophthalmic diagnosis which might reduce the benefit of cataract surgery. One could argue that in other ophthalmology centres the patient sample regarding subjective seeing problems may be more severely affected and that results regarding subjective gain in vision or cost per QALY gained are better. This may certainly be partly true as Finnish cataract operation rates are higher than in many other countries. However, there is great variation in the indications for cataract surgery between many Western countries [29] and it is likely that also in many other Western societies the effectiveness of everyday cataract surgery may not be as good as generally believed. For instance in a large Canadian material, even when measured by a disease specific instrument, 32% of Canadian cataract patients scored higher than 90 points on the 100-point Visual Function Assessment before surgery, and only 70% of patients who underwent cataract surgery experienced improvement [13].

In ophthalmology, generic HRQoL instruments have often shown disappointing treatment results [2,12,13]. This has been interpreted as an insensitivity of the generic instruments to reveal problems related to vision and has led to the development of disease-specific scales. Also in our study, one explanation for the small change observed in the utility score could be the relative insensitivity of the instrument used to measure HRQoL. This, however, is not substantiated by our experience with the 15D instrument in many other routine interventions studied in our sample [15]. Admittedly, the 15D evaluates subjective seeing with only one question whereas disease-specific instruments do this with many more, and are thus capable of distinguishing different aspects of seeing more readily. The question, however, remains whether all different aspects really are important for the everyday life of elderly people, and thus their HRQoL, or are they satisfied even with a less optimal level of seeing. According to some recent studies, the impact of seeing on generic HRQoL may have been exaggerated in the past. Although some studies have established a relationship between trouble of seeing and HRQoL, others have indicated that visual impairment does not affect generic HRQoL as much as generally assumed. For instance in patients with unilateral visual impairment, deteriorated HRQoL as measured by the SF-36 instrument was found only in those moderately to severely affected [30]. In patients with age-related macular disease Espallargues et al. [31], using several HRQoL instruments, reported that even severe visual impairment was not reflected in patients' own assessment of life and satisfaction. Furthermore, also the disease-specific VF-14 score was found to correlate only moderately with visual acuity in the better eye [32].

The calculations concerning the cost per QALY are based on an assumption that utility gained by the operation lasts till the end of life. In some cases this may not be true and, therefore, we may underestimate the true cost per QALY gained. Underestimation may also result from the exclusion of the costs of the visit to the referring ophthalmologist and the follow-up visit for the prescription of eyeglasses. By contrast, since cataract is a progressive disease, we may have underestimated the utility gain and thus overestimated the cost per QALY gained.

In our sample, patients whose both eyes were operated during the 6-month follow-up had the lowest preoperative visual acuity and appeared to gain more from surgery than those who had only one eye operated. This result is in agreement with earlier reports indicating that second-eye surgery may produce better self-assessed visual outcomes and satisfaction than first-eye surgery [33,34]. By contrast, however, our patients who had had their first eye operated before entering this study, did not experience an improvement in HRQoL as a result of second-eye surgery. The optimal sequence and timing, in which the operations are carried out, therefore, still needs investigation.

Impact of cataract surgery on HRQoL in our sample on the whole was low. Part of theoperations can be justifiedas having beenperformed on medical indications to avoid risks of increased intraoperative complicationsbecause of intraocular structures [35], or other possible risksbecause of poor visual acuity [36], or to make possible to follow or treat other intraocular diseases. It is also known that most patients will at some time point need the operation anyway as cataract is a progressive disease leading eventually to major visual disability, and that postponement of surgery may increase the complication rate and diminish the attainable utility gain besides the fact that for many patients extended delay of surgery may cause remarkable disability for a considerable part of their remaining life time [37]. Furthermore, all our study patients entering surgery had objective signs of visual impairment evidenced by poor best-corrected visual acuity in the eye to be operated, which may also be considered a justification for surgery. However, when deciding when to perform an operation, emphasis should also be placed on perceived subjective symptoms and on the subjective benefits the operation may produce for the patient. As Mangione et al. stated [14]: "as physicians attempt to set priorities for the use of elective operations that are designed to preserve the capacity for independent functioning among elderly patients, end points such as physical, social or role functioning may prove to be at least as relevant as traditionally accepted clinical measures of success, such as improvement in Snellen visual acuity after cataract extraction or degrees of range of motion after joint arthroplasty".

## Conclusion

The utility gain observed as the result of routine cataract surgery was small and confined mainly to an improvement in seeing only. The cost per QALY gained was clearly higher than that previously estimated based on registry data. Reasons for the unexpectedly small increase in HRQoL after cataract surgerymay bethattwo thirdsof the patientsreported only minimal preoperative subjective seeing problems despite objective evidence of poor visual acuity in the surgical eye, andone third of patientshad a secondary ophthalmic diagnosis, which mighthave reduced the benefit of cataract surgery. In patients suffering from significant or major preoperative seeing problems, the utility gain was more encouraging. To justify resource use on cataract surgery, the patient has to have definitive medical indications for the surgery or its cost-effectiveness needs to be proven with clear paybacks in the form of improved quality of life. The evaluation of visual acuity or visual quality of life only, although important, serves only the purpose of a health status measure, and is not sufficient to reflect the true HRQoL of patients. Furthermore, patient selection for surgery must be optimised and involve quantifiable subjective measures of seeing, and the custom, in which the operations are carried out (one eye vs. both eyes operated) refined.

## Competing interests

The author(s) declare that they have no competing interests.

## Authors' contributions

PR, KK, HS, and RPR. contributed to the design of the study, analysis of the results and writing of the manuscript. TL and A-M.K. contributed to the analysis of the results and writing of the manuscript. O-PR and MB contributed to the design of the study and writing of the manuscript.
